# Transmission dynamics of HIV-1 subtype B strains in Indonesia

**DOI:** 10.1038/s41598-019-50491-8

**Published:** 2019-09-27

**Authors:** Shuhei Ueda, Adiana Mutamsari Witaningrum, Siti Qamariyah Khairunisa, Tomohiro Kotaki, Kazushi Motomura, Nasronudin, Masanori Kameoka

**Affiliations:** 1 Department of Public Health, Kobe University Graduate School of Health Sciences, Hyogo, Japan; 2 Center for Infectious Diseases, Kobe University Graduate School of Medicine, Hyogo, Japan; 3 Indonesia-Japan Collaborative Research Center for Emerging and Re-emerging Infectious Diseases, Institute of Tropical Disease, Universitas Airlangga, Surabaya, Indonesia; 4 Osaka Institute of Public Health, Osaka, Japan; 5 Faculty of Medicine, Universitas Airlangga, Surabaya, Indonesia; 6 Airlangga Hospital, Surabaya, Indonesia

**Keywords:** Molecular evolution, Viral genetics, HIV infections, Epidemiology, Genetics research

## Abstract

Human immunodeficiency virus type 1 (HIV-1) and acquired immunodeficiency syndrome (AIDS) represent a major public health concern in Indonesia. Although circulating recombinant form (CRF) 01_AE is a predominant subtype in Indonesia, HIV-1 subtype B (HIV-1B) is also widely prevalent. However, the viral genetic evolution, spatial origins, and patterns of transmission of HIV-1B in Indonesia remain unclear. In the present study, we described the evolutionary characteristics and spatial-temporal transmission networks of HIV-1B in Indonesia. To elucidate the epidemiological link between HIV-1B epidemics in Indonesia and those in the remainder of the world, we conducted phylogenetic analyses of HIV-1B strains in Indonesia. Based on the results obtained, at least three epidemic clades [the Indonesia, United States (US), and China clades] of HIV-1B were found to be prevalent in Indonesia. In order to identify the potential source and transmission route of Indonesian HIV-1B strains, we performed Bayesian analyses and constructed Maximum clade credibility trees of each clade. Although some HIV-1B strains in Indonesia were introduced from Thailand, the prevalent HIV-1B strains appeared to have been directly introduced from Europe or America. Indonesian HIV-1B may have spread via the main dispersal of pandemic HIV-1B strains via the US from the Caribbean region rather than being directly introduced from Africa.

## Introduction

Human immunodeficiency virus type 1 (HIV-1) and acquired immunodeficiency syndrome (AIDS) represent a major public health concern in Indonesia. According to UNAIDS data, by the end of 2017, approximately 630,000 (540,000–740,000) people were living with HIV among the total population of 263,510,146 people in Indonesia, with an annual estimated 39,000 cases of AIDS-related death^1^. Although the number of new HIV infections each year has been decreasing in Indonesia, it still remains high, with approximately 49,000 (43,000–57,000) being reported in 2017^1^.

HIV-1 subtype B (HIV-1B) is one of the widest spread subtypes of HIV-1 globally and is also prevalent in Asia. HIV-1B is the most prevalent subtype in some countries in Asia, such as Japan, the Philippines, and Myanmar^2,3^. Furthermore, previous studies reported that HIV-1B was the second most prevalent subtype in other Asian countries, such as Malaysia, China, and Singapore^3–6^. Thus, HIV-1B has played an important role in the spread of HIV-1 in Asian countries. However, information on HIV-1B dynamics in Asia remains limited.

We previously revealed that although circulating recombinant form (CRF) 01_AE is a predominant subtype in Indonesia, HIV-1B is also widely prevalent in this country^7–12^. Eastern parts of Indonesia, such as the Papua and West Papua provinces, have a high prevalence of HIV-1B^7,8,13^. Indonesia is the most epidemic country for HIV in South-East Asia and may largely contribute to the spread of HIV-1 in Asia;^1^ however, the viral genetic evolution, spatial origins, and patterns of transmission of HIV-1B in Indonesia remain unclear. In the present study, we described the evolutionary characteristics and spatial-temporal transmission networks of HIV-1B in Indonesia.

## Results

### Indonesian HIV–1B strains were classified into three distinct clades

**Figure 1:**
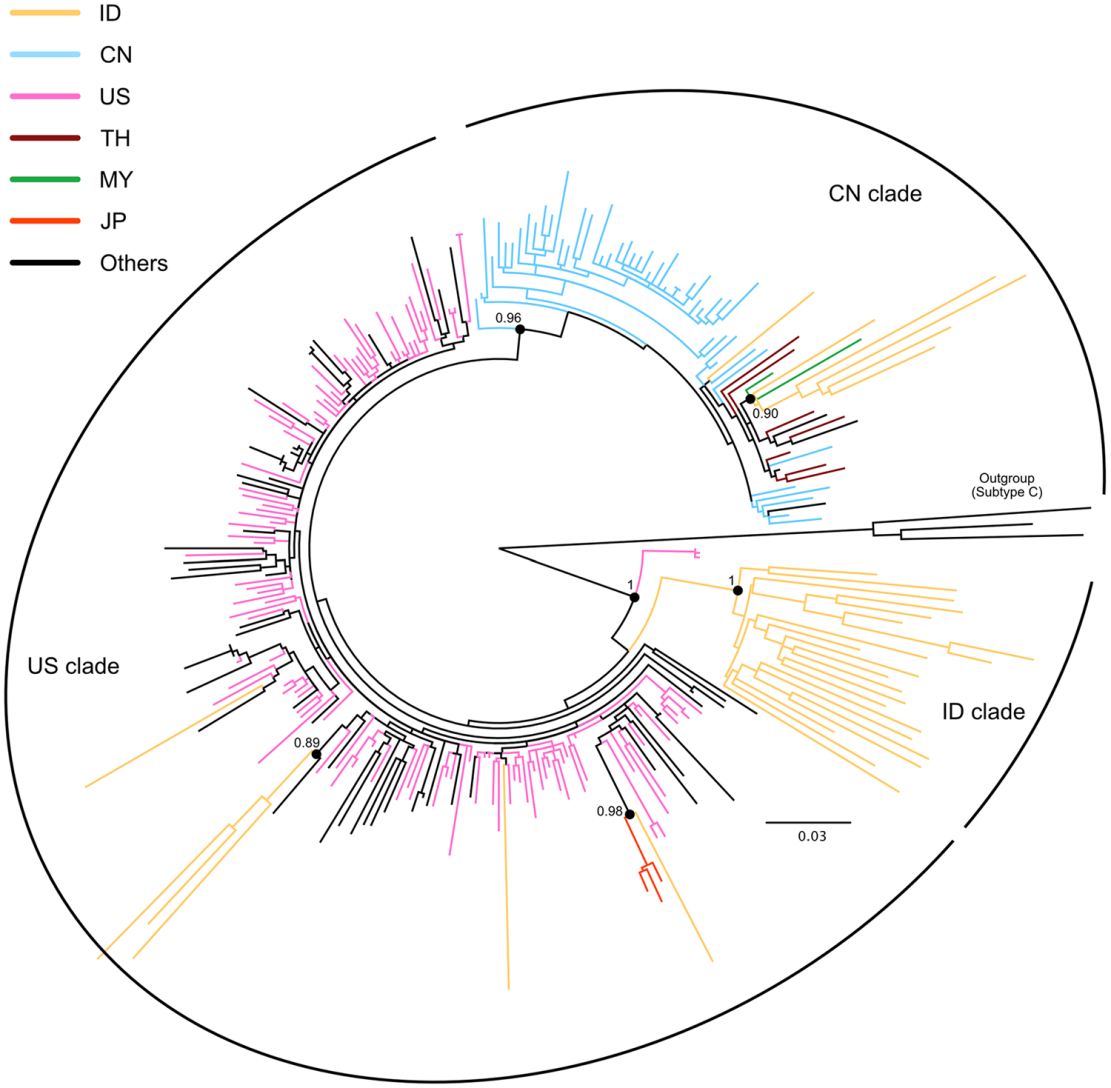
Phylogenetic analysis of HIV-1B in Indonesia. The phylogenetic tree was constructed using the approximately maximum likelihood method based on the *pol* region (HXB2 numbering: nt 2253–3306) in PhyML. HIV-1 subtype C sequences were used as the outgroup in the rooted tree. The nucleotide substitution model was GTR + Γ + I. Approximate Likelihood Ratio Test (aLRT) values ≥ 0.85 were used to identify the clade. The countries of origin of each sequence are indicated by the ISO 3166 two-letter code (https://www.iso.org/obp/ui/#iso:pub:PUB500001:en) as follows: CN, China; ID, Indonesia; US, the United States of America; TH, Thailand; MY, Malaysia; JP, Japan.

To elucidate the epidemiological link between HIV-1B epidemics in Indonesia and those in the remainder of the world, we conducted phylogenetic analyses using our previously identified HIV-1B sequences^7–12^, other reported HIV-1B sequences in Indonesia, and closely related HIV-1B sequences available on the HIV Sequence Database of the Los Alamos National Laboratory (LANL, https://www.hiv.lanl.gov/). HIV-1 subtyping was performed on the *gag*, *pol*, and *env* genes using the Recombination Identification Program (RIP) available on the LANL database in our previous studies^7–12^, and recombinant viruses containing HIV-1B gene fragments were not included in the present study. As Indonesian HIV-1B strains, 32 sequences from our previous studies (Genbank accession no. KM212841, KM212867, KM212896, KM212925, KU596470, KU596473, KU596476, KU596479, KU596480 - KU596482, KU596487, KU596495, KU596498, KU596500, KU596502, KU596507, KU596511, KU596514, KU596517, KU596519 - KU596522, KU596527, KU596534, KU596535, KU596537, KU596538, KU596540, KX639376, KX639377, KX639381, KX639383, KX639384, KX639390, KX639391, KX639395, KX639397, KX639398, MG717595, MG717596, MG717603, MG717604, MG717612, MG717620, MG717631, MG717632, MG717637, MG717638, MG717644, MG717650, MG793084, MG793091, MG793095, MG793097, MG793174, MG793177, MG793183, MG793184, MH727252, and MH727285, HIV-1 *pol* gene sequences encoding viral protease and reverse transcriptase, were separately registered to Genbank)^7–12^ and an Indonesian HIV-1B sequence (GenBank accession no. KY927962) from the LANL database were examined in the present study. Phylogenetic analyses clearly identified three distinct major clades: the Indonesia (ID), United States (US), and China (CN) clades (Fig. 1). Sixty-four percent (21/33) of Indonesian samples belonged to the ID clade, 18% (6/33) to the US clade, and 18% (6/33) to the CN clade. All Indonesian strains that belonged to the ID clade formed a monophyletic clade. Some strains in the US clade also formed a unique sub-clade (Fig. 1). These strains showed similarities to North American strains. Indonesian strains that belonged to the CN clade showed similarities to Chinese, Malaysian, and Thai strains. Most strains in the CN clade also formed a unique sub-clade (Fig. 1).

### Timescales of Indonesian HIV-1B

**Figure 2:**
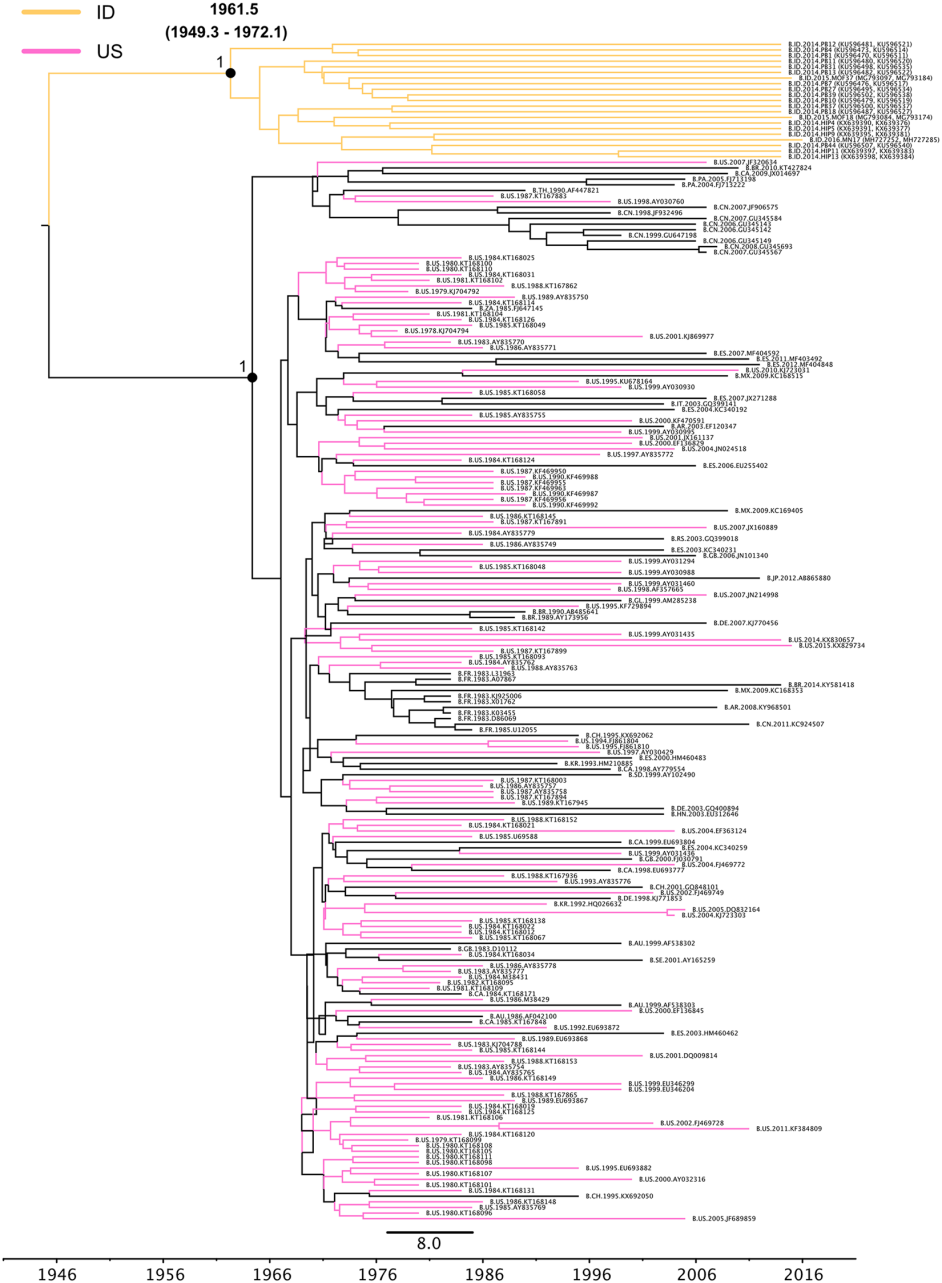
Maximum clade credibility (MCC) phylogenetic tree of *pol* sequences based on the ID clade. The MCC tree of HIV-1B in the ID clade was estimated by a Bayesian Markov chain Monte Carlo approach. Times to the most recent common ancestors (tMRCAs) for phylogenetic clades of interest are indicated as mean dates and 95% highest posterior density (HPD) regions. The geographic origins of the sequences are represented by colors or two-letter country codes as follows: AR, Argentina; AU, Australia; BR, Brazil; CA, Canada; CH, Switzerland; CN, China; DE, Germany; ES, Spain; FR, France; GB, United Kingdom of Great Britain and Northern Ireland; GL, Greenland; HN, Honduras; ID, Indonesia; IT, Italy; JP, Japan; KR, Korea; MX, Mexico; PA, Panama; PH, Philippines; RS, Serbia; SE, Sweden; SD, Sudan; TH, Thailand; US, the United States of America; ZA, South Africa.

Previous studies reported that HIV-1B strains spread globally from West-Central Africa over North America/Western Europe^14,15^. In order to identify the potential source and transmission route of Indonesian HIV-1B strains, we performed Bayesian analyses and constructed maximum clade credibility (MCC) trees of the ID, US, and CN clades. The ID clade represented the only large monophyletic clade with Indonesian sequences in the maximum likelihood tree (Fig. 1). All Indonesian strains in the ID clade also formed one sub-clade in the Bayesian tree (Fig. 2). According to the calculation on posterior probability including estimated times to the most recent common ancestors (tMRCAs), we estimated that these HIV-1B strains were introduced in Indonesia in 1961.8 (1949.2–1972.2) (Fig. 2). Since these strains branched the most rooted position (Fig. 2), the transmission dynamics of Indonesian strains in the ID clade were still unknown based on this result. Furthermore, the tMRCA of Indonesian strains in the ID clade was estimated to be earlier than that of the initial spread of epidemic strains outside Africa in other studies^14,15^. ID clade strains did not show similarities to African strains. Thus, we also conducted a Bayesian analysis using *pol* region sequences without residues that were reported to have drug resistance-associated mutations in order to exclude the impact of antiretroviral drug pressure^16,17^. To this end, we excluded nucleotides (nt)(triplets) for amino acid residues 46, 47, 48, 50, 54, 58, 74, 76, 82, 83, 84, 88, and 90 of protease-coding regions that are related to drug resistance-associated major mutations against protease inhibitors^17^. However, the result obtained was similar to the findings of the original analysis that included entire *pol* regions (data not shown), indicating that antiretroviral drug pressure had no effect on the uniqueness of the *pol* region in ID clade strains.

**Figure 3:**
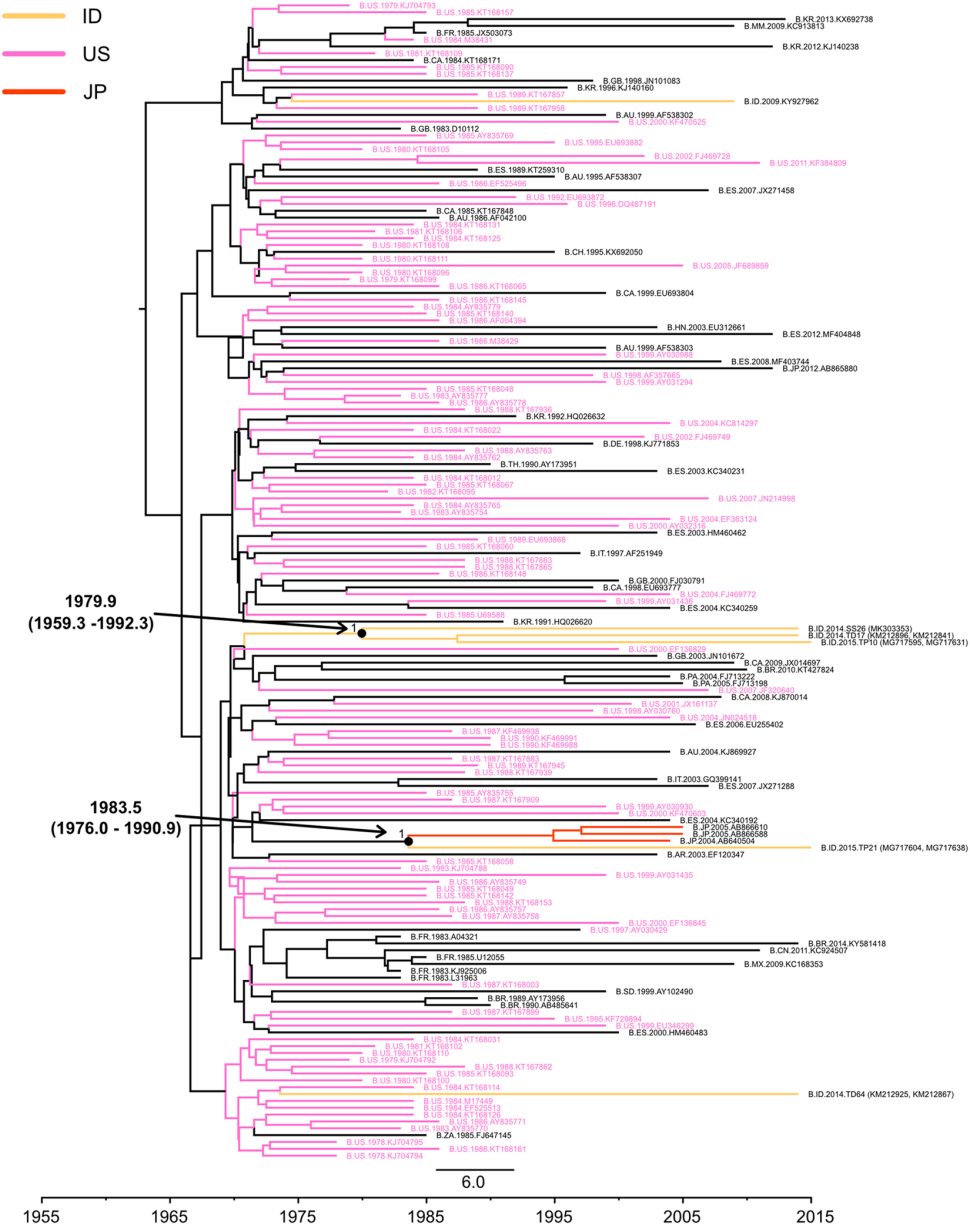
MCC phylogenetic tree of *pol* sequences based on the US clade. The MCC tree of HIV-1B in the US clade was estimated by a Bayesian Markov chain Monte Carlo approach, as described in the legend of Fig. 2. The geographic origins of the sequences are represented by colors or two-letter country codes as follows: AR, Argentina; AU, Australia; BR, Brazil; CA, Canada; CH, Switzerland; CN, China; DE, Germany; ES, Spain; FR, France; GB, United Kingdom of Great Britain and Northern Ireland; HN, Honduras; ID, Indonesia; IT, Italy; JP, Japan; KR, Korea; MM, Myanmar; MX, Mexico; PA, Panama; SD, Sudan; TH, Thailand; US, the United States of America; ZA, South Africa.

**Figure 4:**
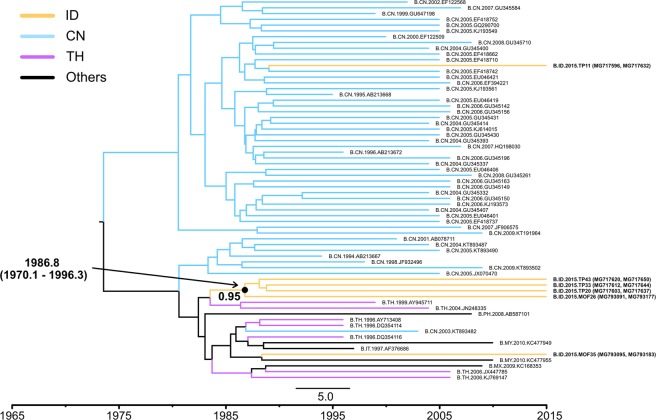
MCC phylogenetic tree of *pol* sequences based on the CN clade. The MCC tree of HIV-1B in the CN clade was estimated by a Bayesian Markov chain Monte Carlo approach, as described in the legend of Fig. 2. The geographic origins of the sequences are represented by colors or two-letter country codes as follows: CN, China; ID, Indonesia; IT, Italy; MY, Malaysia; MX, Mexico; PH, Philippines; TH, Thailand.

Some strains in the US clade formed a sub-clade (Fig. 3). The tMRCA of this sub-clade was estimated to be 1979.9 (1959.3–1992.3). Only one strain (TP21) formed a subclade with Japanese strains, the tMRCA of which was estimated to be 1983.5 (1976.0–1990.9) (Fig. 3). As shown in Fig. 4, most Indonesian strains in the CN clade formed one sub-clade. We estimated that Indonesian strains in the CN clade were introduced in 1986.8 (1970.1–1996.3). Since Indonesian strains in the CN clade were similar to Thai strains, we conducted the Bayesian analysis again including all available Thai HIV-1B sequences (Supplementary Fig. S1). The results obtained revealed that Indonesian strains in the CN clade showed similarities to minor Thai strains that were distinct from major Thai HIV-1B strains.

### Phylogenetic analysis of ID and US clades

**Figure 5:**
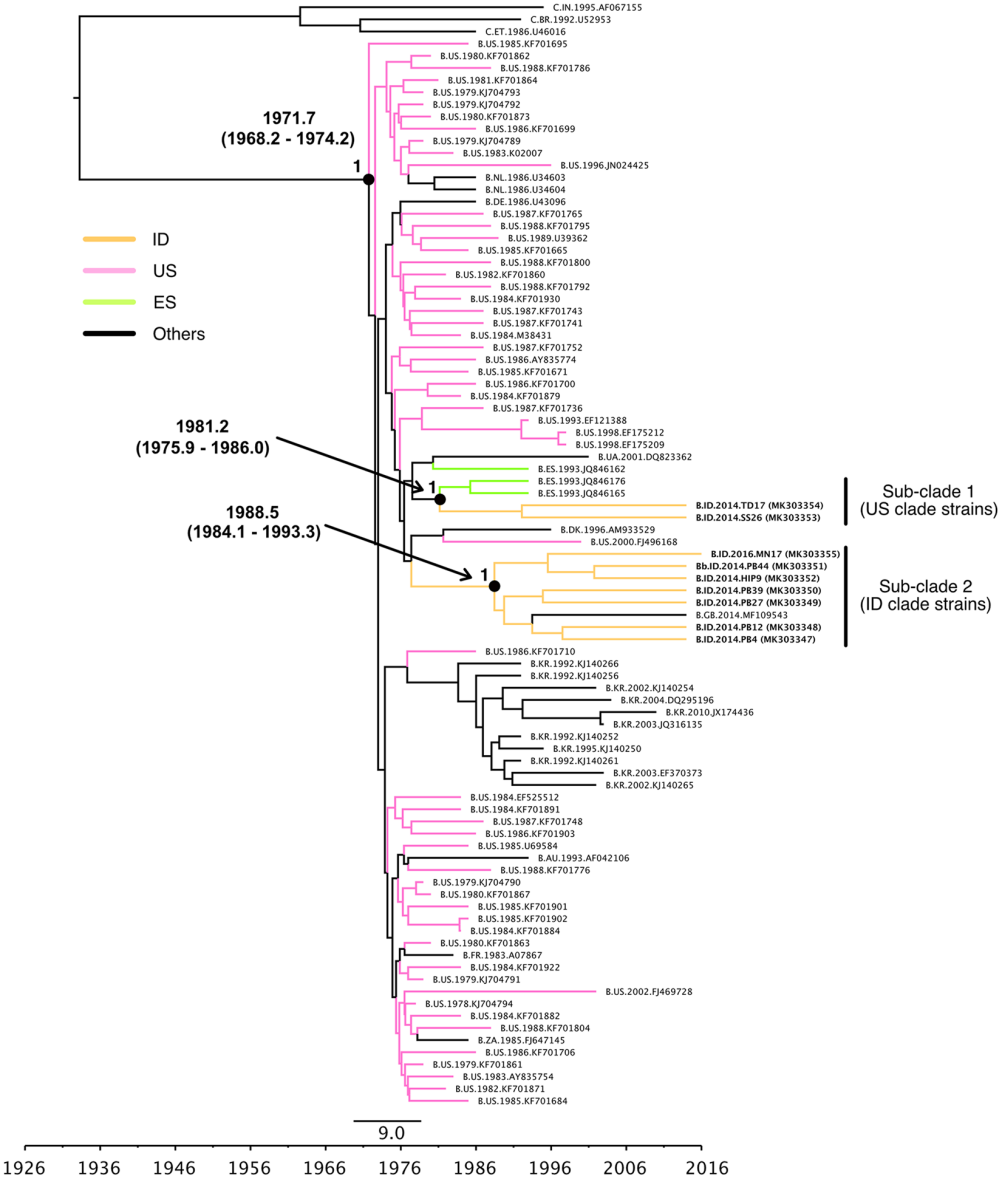
MCC phylogenetic tree of *gag* sequences based on ID and US clades. The MCC tree of HIV-1B of the *gag* region in the ID and US clades was estimated by a Bayesian MCMC approach. HIV-1 subtype C sequences were used as the outgroup in the rooted tree. tMRCAs for phylogenetic clusters of interest are indicated as mean dates and 95% HPD regions. The geographic origins of the sequences are represented by colors or two-letter country codes as follows: AU, Australia; BR, Brazil; DE, Germany; DK, Denmark; ET, Ethiopia; ES, Spain; FR, France; GB, United Kingdom of Great Britain and Northern Ireland; ID, Indonesia; IN, India; KR, Korea; NL, Netherlands; UA, Ukraine; US, the United States of America; ZA, South Africa.

**Figure 6:**
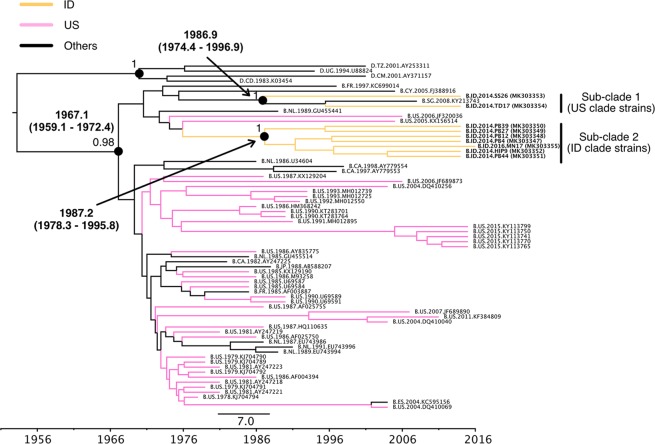
MCC phylogenetic tree of *env* sequences based on ID and US clades. The MCC tree of HIV-1B of the *env* region in the ID and US clades were estimated by a Bayesian MCMC approach, as described in the legend of Fig. 5. The geographic origins of the sequences are represented by colors or two-letter country codes as follows: CA, Canada; CD, Congo; CM, Cameroon; CY, Cyprus; ES, Spain; FR, France; ID, Indonesia; JP, Japan; NL, Netherlands; SG, Singapore; TZ, Tanzania; UG, Uganda; US, the United States of America.

Although the transmission dynamics of the US and CN clades were clearly shown in the *pol* region tree, those of the ID clade remained unclear from the *pol* region tree. In order to identify the potential source and transmission route of Indonesian HIV-1B in the ID clade, we conducted nearly full-genome sequencing and phylogenetic analyses of the *gag* and *env* regions. Since ID clade strains showed similarities to American strains, we also conducted nearly full-genome sequencing of US clade strains and included them in phylogenetic analyses. Nearly full-genome sequencing was successfully conducted on 9 strains in the ID and US clades (TD17, SS26, MN17, PB4, PB12, PB27, PB39, PB44, and HIP9). Full-length *gag* and *env* sequences were then used in phylogenetic analyses. Through Bayesian phylogenetic analyses, we constructed two MCC trees for the *gag* and *env* regions of the ID and US clades (Figs 5 and 6). As shown in the MCC tree of the *gag* region (Fig. 5), US and ID clade strains were separated into sub-clades 1 and 2, respectively. The tMRCAs of subtype B of the ID and US clades and sub-clades 1 and 2 were 1971.7 (1968.2–1974.2), 1981.2 (1975.9–1986.0), and 1988.5 (1984.1–1993.3), respectively (Fig. 5). Sub-clade 1 strains showed similarities to Spanish strains, while sub-clade 2 strains showed similarities to the American and Denmark strains. In addition, one strain from the United Kingdom was included in sub-clade 2.

According to the *env* tree (Fig. 6), Indonesian HIV-1B strains were also separated into two types of sub-clades. Similar to the *gag* tree, sub-clade 1 only included US clade strains, while sub-clade 2 only included ID clade strains (Fig. 6). The tMRCAs of HIV-1B of the ID and US clades and sub-clades 1 and 2 were 1967.1 (1959.1–1972.4), 1986.9 (1974.4–1996.9), and 1987.5 (1978.3–1995.8), respectively (Fig. 6). US clade strains (sub-clade 1) were similar to a Cyprus strain, and a Singaporean strain was included in this sub-clade (Fig. 6). ID clade strains (sub-clade 2) were similar to US strains and this sub-clade only formed Indonesian strains (Fig. 6). The estimated tMRCAs at the root of HIV-1B in the *gag* and *env* regions were consistent with previous findings^14,15^. Based on *gag* and *env* analyses, US clade strains were estimated to have been introduced from European countries in the early or mid-1980s, while ID clade strains were introduced from America in the late 1980s.

## Discussion

HIV infection is one of the most important public health concerns in Indonesia, the fourth most populous country in the world. HIV-1B is the second most prevalent subtype, although CRF01_AE is predominant in Indonesia. CRF01_AE is a major CRF circulating in South-East Asian countries including Indonesia and Thailand^3^. According to our previous study, Indonesian CRF01_AE strains have a phylogenetically close relationship with Thai CRF01_AE strains (unpublished). In contrast, the viral genetic evolution, spatial origins, and patterns of transmission of HIV-1B in Indonesia remain unclear. In the present study, we revealed some of the transmission dynamics of HIV-1B in Indonesia. Based on the present *pol* region study (Fig. 1), at least three epidemic clades (the ID, US, and CN clades) of HIV-1B were found to be prevalent in Indonesia. There were more Indonesian strains in the ID and US clades than in the CN clade, and ID and US clade strains spread to many islands in Indonesia. ID clade strains were found in Papua, Sulawesi, and Flores islands, while US clade strains were detected in Jawa and Sumatra islands. Thus, ID and US clade strains appear to be the most prevalent HIV-1B strains in Indonesia.

Most Indonesian strains in the ID clade formed a unique clade (Fig. 2); therefore, it was impossible to elucidate transmission dynamics from the Bayesian analysis in the *pol* region. The estimated tMRCA of Indonesian strains in the ID clade was earlier than that of the initial spread of the epidemic outside of Africa in other studies^14,15^. Furthermore, antiretroviral drug pressure did not appear to affect the uniqueness of the *pol* region in ID clade strains (data not shown). Although the reason why most Indonesian strains formed a distinct clade in the *pol* region currently remains unknown, we propose the following. HIV-1B strains in the ID clade may have emerged earlier than or in parallel with the global spread of HIV-1B^14,15^ and have evolved uniquely in Indonesia. The overall mean pairwise distances of the *pol* genes derived from the ID, CN, and US clade strains were calculated to be 0.057, 0.062, and 0.075, respectively, by the standard function of MEGA 7.0.21, indicating that the overall genetic distance of the ID clade strains was slightly lower than those of the CN and US clade strains. Thus, we assumed that ID clade strains were a transmission cluster, and analyzed the individual data of study participants. Regarding the results obtained, samples were collected from males (42%) or females (58%) of various ages at Papua, Sulawesi, or Flores islands between 2014 and 2016 (data not shown). According to the questionnaire survey, possible transmission routes were heterosexual transmission for most samples, except for one sample of mother-to-child transmission (data not shown). Therefore, ID clade strains may have uniquely evolved and spread by heterosexual transmission to different areas of Indonesia. In order to clarify the uniqueness of the distinct monophyletic ID clade, it is important to accumulate more sequence information from Asian countries and Indonesia.

To elucidate the transmission dynamics of Indonesian strains in the ID and US clades, we conducted nearly full-genome sequencing and Bayesian analyses of the *gag* and *env* regions (Figs 5 and 6). Based on the results obtained, US clade strains (sub-clade 1) were estimated to have been introduced from Europe in the early or mid-1980s. This introduction date was similar to those of other Asian countries, such as Japan and China^16,18,19^. The results of ID clade strains (sub-clade 2) in the *gag* and *env* regions suggest that ID clade strains were introduced from America in the late 1980s. In the *gag* and *env* trees, sub-clades 1 and 2 included a Singaporean strain and United Kingdom strain, respectively. This result suggests that HIV-1B was imported into Indonesia from other countries, and HIV-1B in Indonesia was then exported to other Asian and European countries. Two US clade strains (sub-clade 1), TD17 and SS26, were collected in Jawa island, while 7 ID clade strains (sub-clade 2) were collected in Sulawesi (MN17) and Papua islands (PB4, PB12, PB27, PB39, PB44, and HIP9), indicating that these strains were circulating in the central, midwestern, and western parts of Indonesia.

Indonesian HIV-1B in the CN clade was estimated to have been introduced from Thailand in the late 1980s. A previous study revealed that the tMRCA of the Thai subtype B variant in China was estimated to be in the early 1980s^20^. Thus, CN clade strains in Indonesia appear to have been introduced into Indonesia at the same time or slightly later than their introduction into China. HIV-1B in the CN clade was similar to minor Thai strains (Supplementary Fig. 1S). Further genome information needs to be collected in South-East Asian countries in order to elucidate the relationship between these strains in more detail. Since HIV-1B in the CN clade formed a unique sub-clade, these strains may circulate domestically and have uniquely evolved in Indonesia. All viral strains in the CN clade were collected in the Riau (TP11, TP20, TP33, and TP43) and East Nusa Tenggara provinces (MOF26 and MOF35). These provinces are on different islands (Sumatera and Flores islands, respectively) and are not geographically close. Thus, these strains appear to have spread widely in Indonesia, while only a small number of CN clade strains was detected.

The present study had the following limitations. Thirty-three Indonesian HIV-1B gene sequences were studied, and the sample size may not have been sufficiently large to provide clear research outcomes. However, HIV-1B samples were a minority in Indonesia, and were found in less than 20% of samples, even including Papua and West Papua samples, in our previous studies^7–12^. Furthermore, although a high prevalence of HIV-1B was detected in the Papua and West Papua provinces^7,8,13^, difficulties were associated with performing field studies on these provinces. Therefore, the collection of more HIV-1B samples has not proceeded efficiently in Indonesia. Nevertheless, we consider the present results to provide an insight into the origin of Indonesian HIV-1B. In addition, in order to conduct phylogenetic analyses, we retrieved HIV-1B gene sequences closely related to Indonesian strains by a BLAST search (https://blast.ncbi.nlm.nih.gov/Blast.cgi). Regarding the results obtained, viral genes isolated in different countries were selected for each phylogenetic analysis. This approach may lead to discrepant study outcomes among the *gag*, *pol*, and *env* genes of the ID clade strains. However, we considered it important to analyze phylogenetically close viral sequences, and adopted this approach in the present study.

**Figure 7:**
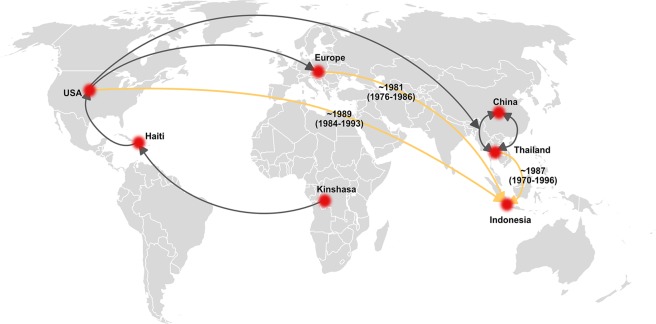
Estimated spatial dynamics of HIV-1B in Indonesia. Gray and orange lines represent the estimated dissemination routes of HIV-1B in other studies^14,27^ and in the present study, respectively. The epidemic started in the city of Kinshasa, the Democratic Republic of the Congo. HIV-1B then spread through Haiti and America. There are 3 different HIV-1B clades in Indonesia (the ID, CN, and US clades). The estimated tMRCAs (95% HPD) analyzed in the *gag* region for the ID and US clades and in the *pol* region for the CN clade are shown.

In conclusion, HIV-1B was introduced several times into Indonesia from Thailand, Europe, and America. Although some HIV-1B strains in Indonesia were also introduced from Thailand, the prevalent HIV-1B strains in Indonesia appeared to be directly introduced from Europe or America (Fig. 7) and some were exported to Asian and European countries. Indonesian HIV-1B may have spread by the global dispersal of pandemic HIV-1B strains via the US from the Caribbean region rather than being directly introduced from Africa.

## Methods

### Sequence data

HIV-1B *pol* sequences (protease 1–99 and reverse transcriptase 1–250 amino acids) from our previous research in Indonesia^7–12^ and available sequences downloaded from the HIV Sequence Database of LANL were included in the present study. Thirty-three HIV-1B *pol* sequences covering 1054 base pairs [corresponding to nt 2253–3306 of the HIV-1 reference strain, HXB2 (GenBank accession no. K03455) (HXB2 numbering: nt 2253–3306)] were studied^6,21^.

Each of the 25 HIV-1B *pol* sequences (HXB2 numbering: nt 2253–3306) closely related to 33 Indonesian strains were selected by a BLAST search and downloaded from the HIV Sequence Database. A total of 242 reference sequences with the highest similarities to all Indonesia sequences were selected after manually removing closely related sequences from the same areas or sequences with no information on collection dates and regions. We compared the outcomes of phylogenetic analyses (described below) with and without including sequences with no information on collection dates; however, the results obtained were similar (data not shown). The database included 275 HIV-1B *pol* sequences for phylogenetic analyses. The accession numbers of downloaded sequence data are available upon request.

### Phylogenetic analysis

A maximum likelihood tree was constructed using the general time reversible (GTR) + Γ + I model in PhyML^22^ in order to elucidate phylogenetic interrelationships among viral sequences. The phylogenetic tree’s reliability was evaluated using the approximate Likelihood Ratio Test (aLRT) of SH-like supports^23^. The final tree was generated using FigTree v1.4.3. Monophyletic groups with aLRT support >0.85 were considered to be a clade.

### Bayesian phylogenetic analyses

Bayesian phylogenetic analyses were conducted using BEAST v.1.8.4 for the ID, US, and CN clades. We conducted a BLAST search for the ID clade. We initially compared the setting of BEAST, such as the substitution model: HKY or GTR, clock model: uncorrelated lognormal or uncorrelated exponential, and tree prior: constant size or Bayesian skyline. To this end, we performed more than 8 computations and compared effective sample size (ESS) values. Regarding the results obtained, a setting with the combination of the substitution model: GTR, clock model: uncorrelated lognormal, and tree prior: Bayesian skyline showed the highest ESS value. Thus, model compositions were performing Bayesian analyses of the GTR substitution model and an uncorrelated lognormal relaxed molecular clock model for 100 million generations for the CN clade and 200 million generations for the ID and US clades with sampling every 1000 steps. An ESS value > 200 was interpreted as a convergence of the Markov chain Monte Carlo (MCMC) sample on the posterior distribution after discarding the first 10% as a burn-in. MCC trees summarizing the posterior distribution were generated with TreeAnnotator and visualized in FigTree v1.4.3. The same database controls were used in the Maximum likelihood and Bayesian analyses.

### Nearly full-genome sequencing

We conducted nearly full-genome sequencing on Indonesian HIV-1B of the ID and US clades (TD17, SS26, MN17, PB4, PB12, PB27, PB39, PB44, and HIP9). DNA was extracted from the peripheral blood samples of HIV-1-infected individuals using the QIAamp DNA blood mini kit (Qiagen), as described previously^7–12^. The nearly full-length HIV-1B genome was then amplified in two fragments. The 5′ terminal half of the HIV-1 genome (former fragment) and 3′ terminal half of the genome (latter fragment) were individually amplified by nested PCR using the Expand Long Template PCR System (Roche Diagnostics). Regarding the former fragment, first-round PCR was performed with the msf12b^24,25^ and DRIN02 primers^26^ and was followed by second-round nested PCR with the f2nst^24^ and DRIN04 primers^26^. The conditions for PCR were as follows: in first PCR, initial denaturation at 94 °C for 2 minutes followed by 10 cycles of 94 °C for 10 seconds, 55 °C for 30 seconds, and 68 °C for 4 minutes, and 25 cycles of 94 °C for 15 seconds, 55 °C for 30 seconds, and 68 °C for 4 minutes and 20 seconds, and final extension at 68 °C for 7 minutes. In the latter cycles, elongation times were extended for 20 seconds for each cycle. In second PCR, 50 °C was used as the annealing temperature. Regarding the latter fragment, first-round PCR was performed with the DRIN01^26^ and UNINEF’7^24^ primers and was followed by second-round nested PCR with the DRIN05^26^ and nefyn05 primers^24^. The conditions for PCR were as follows: in first PCR, initial denaturation at 94 °C for 2 minutes followed by 10 cycles of 94 °C for 15 seconds, 54.5 °C for 30 seconds, and 68 °C for 6 minutes, and 25 cycles of 94 °C for 15 seconds, 54.5 °C for 30 seconds, and 68 °C for 6 minutes and 20 seconds, and final extension at 68 °C for 7 minutes. In the latter cycles, elongation times were extended for 20 seconds for each cycle. In second PCR, 62 °C was used as the annealing temperature. Sanger sequencing of the successfully amplified former and latter fragments was then performed using the BigDye Terminator version 3.1 Cycle Sequencing kit and ABI PRISM3500Xl genetic analyzer (Applied Biosystems). Information on sequencing primers is available upon request. Sequenced fragments were assembled into contiguous sequences using Genetyx ver. 10 software (Genetyx). The nucleotide sequences of the nearly full-genomes have been registered in the GenBank database under accession numbers MK303347-MK303355.

### Phylogenetic analysis of *gag* and *env* regions

All 25 closely related HIV-1B *gag* and *env* sequences (HXB2 numbering: nt 790–2292 for *gag* and nt 6225–8795 for *env*) were downloaded from the HIV Sequence Database of the LANL after a BLAST search against the 9 Indonesia HIV-1B sequences. The resulting datasets were aligned and then adjusted by eye in MEGA 7.0.21 and regions of ambiguous alignment were removed. Bayesian phylogenetic analyses were then conducted using the same models for the HIV-1B *pol* analysis.

### Ethics statement

Ethical clearance was obtained from the Institutional Ethics Committees of Universitas Airlangga (approval number: 25–995/UN3.14/PPd/2013) and Kobe University Graduate School of Medicine (approval number: 784). Written informed consent was obtained from all study participants from whom peripheral blood DNA samples were collected. All experiments were performed in accordance with the relevant guidelines and regulations.

## Supplementary information

Supplementary Figure S1

## Data Availability

All necessary data generated or analyzed during the present study are included in this published article and its Supplementary Information files.
