# Supplementary material for: Transmission dynamics of HIV-1 subtype B strains in Indonesia
Source: Sci Rep. 2019 Sep 27;9:13986. doi: 10.1038/s41598-019-50491-8 (PMC6764962; doi:10.1038/s41598-019-50491-8)
Supplement: Supplementary file 1 — Supplementary Figure S1 [file 41598_2019_50491_MOESM1_ESM.pdf]

Supplementary Information

Transmission dynamics of HIV-1 subtype B strains in Indonesia

Shuhei Ueda,[1,2,3] Adiana Mutamsari Witaningrum,[3] Siti Qamariyah Khairunisa,[3] Tomohiro Kotaki,[1,3] Kazushi Motomura,[4] Nasronudin,[3,5,6] and Masanori Kameoka[1,2,*]

[1]Department of Public Health, Kobe University Graduate School of Health Sciences, Hyogo, Japan
[2]Center for Infectious Diseases, Kobe University Graduate School of Medicine, Hyogo, Japan
[3]Indonesia-Japan Collaborative Research Center for Emerging and Re-emerging Infectious Diseases, Institute of Tropical Disease, Universitas Airlangga, Surabaya, Indonesia
[4]Osaka Institute of Public Health, Osaka, Japan
[5]Faculty of Medicine, Universitas Airlangga, Surabaya, Indonesia
[6]Airlangga Hospital, Surabaya, Indonesia

Corresponding author: Masanori Kameoka
Department of Public Health, Kobe University Graduate School of Health Sciences,
7-10-2 Tomogaoka, Suma-ku, Kobe, Hyogo 654-0142, Japan
Tel./Fax: +81-78-796-4594
E-mail: mkameoka@port.kobe-u-ac.jp

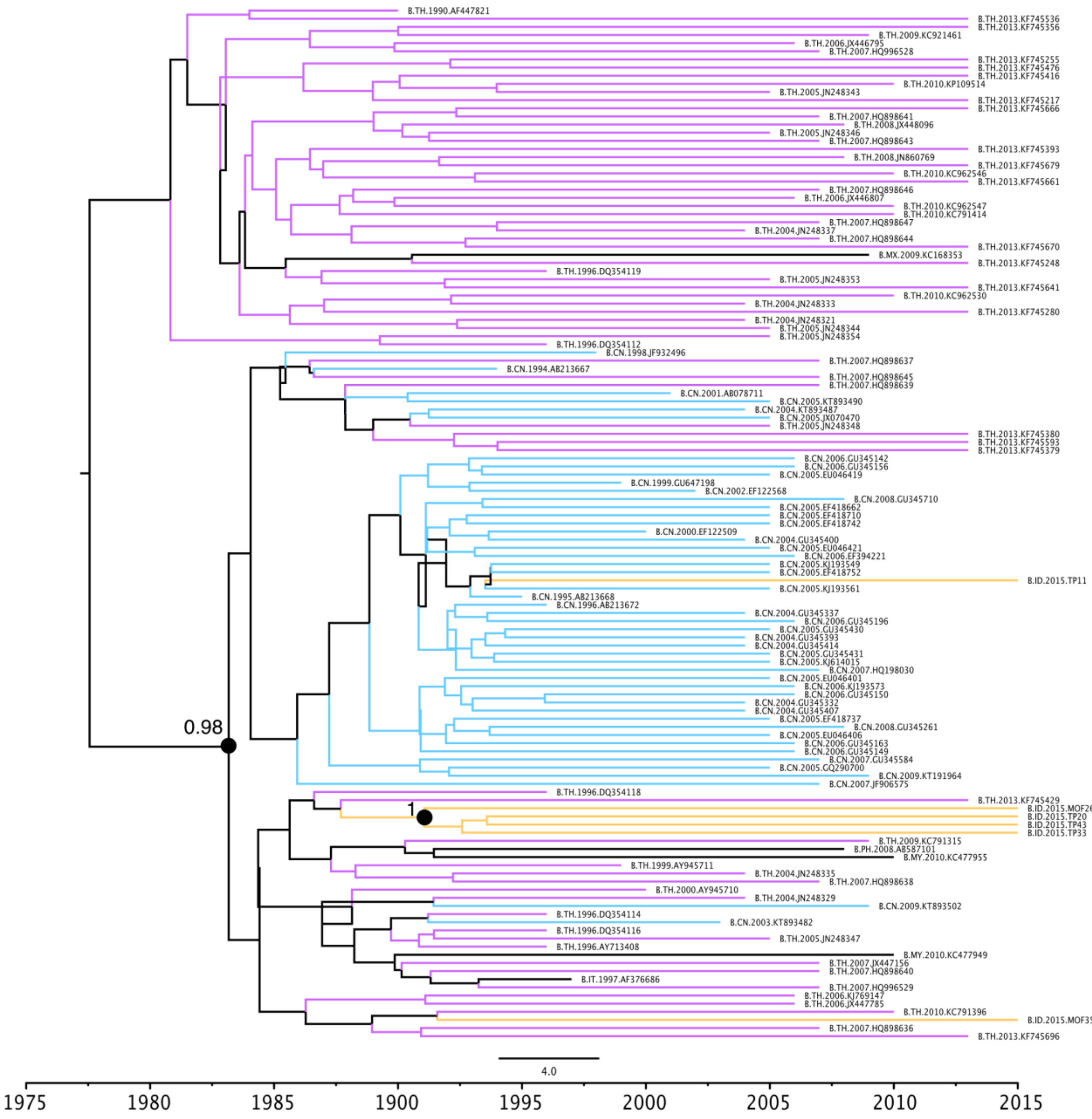

Supplementary Figure S1. MCC phylogenetic tree of *pol* regions based on the CN clade after the addition of all available Thai strains.

All available Thai strains were added to CN clade strains. The MCC tree of HIV-1B was estimated by a Bayesian MCMC approach. Greater than or equal to 0.9 of posteriori probability was shown in the tree. The geographic origins of the sequences are represented by colors or two-letter country codes as follows: CN, China; ID, Indonesia; IT, Italy; MY, Malaysia; MX, Mexico; PH, Philippines; TH, Thailand.
